# A Prospective Study Evaluating Cumulative Incidence and a Specific Prediction Rule in Pulmonary Embolism in COVID-19

**DOI:** 10.3389/fmed.2022.936816

**Published:** 2022-07-01

**Authors:** Carla Suarez Castillejo, Nuria Toledo-Pons, Néstor Calvo, Luisa Ramon-Clar, Joaquín Martínez, Sara Hermoso de Mendoza, Daniel Morell-García, Josep Miquel Bauça, Francisco Berga, Belén Núñez, Luminita Preda, Jaume Sauleda, Paula Argente Castillo, Antonieta Ballesteros, Luisa Martín, Ernest Sala-Llinas, Alberto Alonso-Fernández

**Affiliations:** ^1^Servicio de Neumología, Hospital Universitario Son Espases, Palma de Mallorca, Spain; ^2^Institut d’Investigació Sanitària Illes Balears (IdISBa), Palma, Spain; ^3^Servicio de Radiodiagnostico, Hospital Universitario Son Espases, Palma de Mallorca, Spain; ^4^Complejo Hospitalario de Navarra, Pamplona, Spain; ^5^Servicio de Análisis Clínicos, Hospital Universitario Son Espases, Palma de Mallorca, Spain; ^6^CIBER Enfermedades Respiratorias, Palma de Mallorca, Spain; ^7^Servicio de Medicina Interna, Hospital Universitario Son Espases, Palma de Mallorca, Spain

**Keywords:** computed tomography angiography, coronavirus infections, fibrin fibrinogen degradation products, pandemics, SARS-CoV-2, venous thromboembolism, clinical decision rule

## Abstract

**Rationale:**

Abnormal values of hypercoagulability biomarkers, such as D-dimer, have been described in Coronavirus Disease 2019 (COVID-19), which has also been associated with disease severity and in-hospital mortality. COVID-19 patients with pneumonia are at greater risk of pulmonary embolism (PE). However, the real incidence of PE is not yet clear, since studies have been limited in size, mostly retrospective, and PE diagnostic procedures were only performed when PE was clinically suspected.

**Objectives:**

(1) To determine the incidence, clinical, radiological, and biological characteristics, and clinical outcomes of PE among patients hospitalized for COVID-19 pneumonia with D-dimer > 1,000 ng/mL. (2) To develop a prognostic model to predict PE in these patients.

**Methods:**

Single-center prospective cohort study. Consecutive confirmed cases of COVID-19 pneumonia with D-dimer > 1,000 ng/mL underwent computed tomography pulmonary angiography (CTPA). Demographic and laboratory data, comorbidities, CTPA scores, treatments administered, and clinical outcomes were analyzed and compared between patients with and without PE. A risk score was constructed from all these variables.

**Results:**

Between 6 April 2020 and 2 February 2021, 179 consecutive patients were included. The overall incidence of PE was 39.7% (71 patients) (CI 95%, 32–47%). In patients with PE, emboli were located mainly in segmental/subsegmental arteries (67%). Patients with PE did not differ from the non-PE group in sex, age, or risk factors for thromboembolic disease. Higher urea, D-Dimer, D-dimer-to-ferritin and D-dimer-to-lactate dehydrogenase (LDH) ratios, platelet distribution width (PDW), and neutrophil-to-lymphocyte ratio (NLR) values were found in patients with PE when compared to patients with non-PE. Besides, lymphocyte counts turned out to be lower in patients with PE. A score for PE prediction was constructed with excellent overall performance [area under the ROC curve-receiver operating characteristic (AUC-ROC) 0.81 (95% CI: 0.73–0.89)]. The PATCOM score stands for *Pulmonary Artery Thrombosis in COVID-19 Mallorca* and includes platelet count, PDW, urea concentration, and D-dimer-to-ferritin ratio.

**Conclusion:**

COVID-19 patients with pneumonia and D-dimer values > 1,000 ng/mL were presented with a very high incidence of PE, regardless of clinical suspicion. Significant differences in urea, D-dimer, PDW, NLR, and lymphocyte count were found between patients with PE and non-PE. The PATCOM score is presented in this study as a promising PE prediction rule, although validation in further studies is required.

## Introduction

The disease caused by the novel severe acute respiratory syndrome coronavirus-2 (SARS-CoV-2) presents heterogeneous clinical features, from asymptomatic infection to acute respiratory distress syndrome, with mortality rates ranging from 0.15 to 5%, and substantial variability by location and certain underlying medical comorbidities (1, 2). Further, fatality rates are higher when D-dimer is > 1,000 ng/mL among hospitalized patients and increase exponentially with age (2–5). Likewise, severe Coronavirus Disease-19 (COVID-19) may show systemic hyper-inflammation (6) and complex coagulation abnormalities that generate a hypercoagulable state (5). Previous studies suggest a possible association between COVID-19 and pulmonary embolism (PE) (7–12). Some risk factors for severe COVID-19 and death, including obesity, increasing age, and a hypercoagulable state, are the same as those for PE. Moreover, patients with COVID-19 are usually bedridden for many days during the acute phase. Rates of PE complications in COVID-19 are low in non-hospitalized patients with asymptomatic or mild diseases but higher with increasing severity of the infection, with those in the intensive care unit (ICU) being at the greatest risk (13, 14). Furthermore, PE has been associated with higher mortality rates (8, 13), and recent data suggest that intermediate-dose thromboprophylaxis is not superior to standard thromboprophylaxis in critically ill patients (14). Nevertheless, elsewhere, a much lower incidence of PE was found among hospitalized patients with COVID-19 (15, 16).

Although the etiopathogenesis of PE in COVID-19 is poorly understood, factors related to the acute inflammatory response to the disease may be contributing to a dysregulation of the equilibrium of procoagulant and anticoagulant mechanisms (2, 6, 17).

Pulmonary embolism diagnosis is a challenging task in COVID-19 hospitalized patients, mainly due to its non-specific clinical presentation and also because symptoms of PE overlap with COVID-19. Nevertheless, early PE diagnosis is essential, as well-timed treatment is highly effective and proven to significantly influence clinical outcomes (18). D-dimer values > 1,000 ng/mL are relatively common in patients with COVID-19, ranging from 40 to 70% (5, 19), with a more pronounced increase among severe cases (3, 20).

In a preliminary study, we prospectively evaluated the incidence of PE in 30 hospitalized patients with COVID-19 pneumonia and D-dimer > 1,000 ng/mL and found an incidence of 50%, regardless of clinical suspicion (12). However, this incidence may have been conditioned by the limited sample size. Additionally, other studies that analyzed the incidence of PE in COVID-19 were of a retrospective nature; diagnostic procedures were only performed when thrombotic complications were suspected, so the reported incidence may not be considered definitive either (7, 8, 21–25). Overall, all the above seem to indicate that high D-dimer values are common in patients with severe forms of COVID-19 and, as such, it is, therefore, reasonable to believe that patients with COVID-19 are at high risk of PE. Accordingly, we aimed to prospectively evaluate the incidence of PE in patients admitted with COVID-19 pneumonia and D-dimer > 1,000 ng/mL, regardless of clinical suspicion. As secondary objectives, we evaluated clinical, radiological, and biochemical variables that could be potentially related to this event; determined whether patients with PE had worse clinical outcomes; and developed a prognostic model to predict PE in this cohort of hospitalized patients with COVID-19.

## Materials and Methods

### Study Design and Participants

A single-center prospective cohort study was performed (Hospital Universitari Son Espases, Palma, Spain). All consecutive adult patients with confirmed COVID-19 pneumonia admitted to the hospital and with at least one D-dimer measurement > 1,000 ng/mL during hospitalization were selected. COVID-19 was confirmed by a positive result on polymerase chain reaction (PCR) testing of a nasopharyngeal sample.

Patients were excluded if they were on anticoagulant treatment for 3 months before admission or were unable to undergo a computed tomography pulmonary angiography (CTPA) for any of the following reasons: unwillingness or inability to participate in the study; allergy to iodinated contrast; or any other concurrent clinical condition which, in the researcher’s opinion, would contraindicate their participation in the study.

All patients were followed up throughout the hospital admission period until hospital discharge or death. The strengthening the reporting of observational studies in epidemiology (STROBE) statement was followed (26).

The sample size was calculated to analyze the estimated incidence of PE in hospitalized patients because of COVID-19 pneumonia with a D-dimer value > 1,000 ng/mL in a previous study, revealing a 50% incidence of PE (12). Assuming a calculation error of 3% and a confidence level of 90%, 179 patients with a valid CTPA scan were needed. The study was completed when the sample size was reached.

### Description of Investigations Undertaken

Epidemiological, demographic, clinical, and laboratory examinations were collected from all subjects at the time of admission. The data recorded included time from symptom onset to hospital admission and to CTPA, medical treatment during hospitalization, thromboprophylaxis, respiratory support, clinical outcomes (acute respiratory failure, arrhythmia, ICU admission, or death), and strong-moderate PE risk factors. The CURB-65 score was calculated (27).

Laboratory data included complete blood count (Cell-Dyn Sapphire platform, Abbott Diagnostics, United States), coagulation, and kidney and liver function tests collected upon admission. In addition, baseline, peak, and prior-to-CTPA values of the following biomarkers were analyzed in each patient: fibrinogen, D-dimer (reported as D-dimer units (DDUs), ACL TOP 700, Instrumentation Laboratory), C-reactive protein (CRP), lactate dehydrogenase (LDH), erythrocyte sedimentation rate (ESR), and ferritin. In addition, high sensitive troponin I, interleukin-6 (IL-6), interleukin-10 (IL-10) (ELISA, R&D systems), N-terminal pro hormone B-type natriuretic peptide (NT pro-BNP) (Test 1 THL Module, ALI FAX; Architect platform, Abbott Diagnostics), and fibrinogen were also measured. Blood gas analyses were performed on the GEM 4000 platform (Werfen, Spain). D-dimer-to-ferritin, D-dimer-to-LDH, and D-dimer-to-CRP ratios were calculated.

### Computed Tomography Pulmonary Angiography

Computed tomography pulmonary angiography was requested per protocol only when the D-dimer determination was > 1,000 ng/mL, regardless of symptoms. Diagnosis of PE was performed by an expert radiologist based on direct visualization of the endoluminal thrombus in the pulmonary arteries. A quantitative assessment of the magnitude of the embolism was calculated with the pulmonary artery obstruction index (PAOI) (28). COVID-19 total lung involvement was automatically calculated by artificial intelligence analysis performed using InferRead™ CT Lung (COVID-19) (Infervision, Europe GmbH, Germany) (29). The right ventricle to left ventricle diameter ratio (RV/LV) was measured as an indicator of RV dysfunction (18).

### Ethics Statement

The Institutional Ethics Committee of the Balearic Islands approved the study (IB 4197/20 PI), and all subjects gave their written informed consent. Only patients with a critical clinical condition gave verbal consent instead, in front of at least two witnesses.

### Statistical Analysis

Descriptive statistics included frequencies and percentages for categorical variables and medians and interquartile ranges (IQRs) for continuous variables. Comparisons were determined by the Mann-Whitney *U*-test for continuous variables, and by the χ^2^-test or Fisher’s exact test for categorical variables. Spearman’s correlation was used to assess relationships between PAOI and biomarkers.

The total population of the study was divided into two groups: patients who either did not require ICU-level care or underwent CTPA before their ICU admission (Group A) and ICU patients who underwent CTPA during or after ICU admission (Group B). Baseline variables related to the presence of PE in the regression analysis were dichotomized to construct predictive scores for PE with patients from Group A. Youden’s index criteria were used to determine the cut-off point for each variable. After dichotomization, these variables were included in a logistic regression model. The beta coefficient of each variable was divided by the smallest absolute value of the regression coefficient and rounded to the nearest integer to calculate the relative weight of each variable. The sum of the relative weight of all the variables included in the score corresponded to its total value. The discriminatory capacity of the score was evaluated by a receiver operating characteristic (ROC) curve analysis, and the sensitivity, specificity, and positive and negative predictive values of different cut-off points were calculated. The calibration and overall performance of the model were evaluated using the Hosmer-Lemeshow goodness-of-fit test (HL) and the Nagelkerke *R*^2^ score, respectively. Differences were considered statistically significant at a 2-tailed *p*-value of < 0.05. The statistical software used was SPSS v.26 (IBM Corporation, United States).

## Results

### COVID-19 Pneumonia Population

**FIGURE 1:**
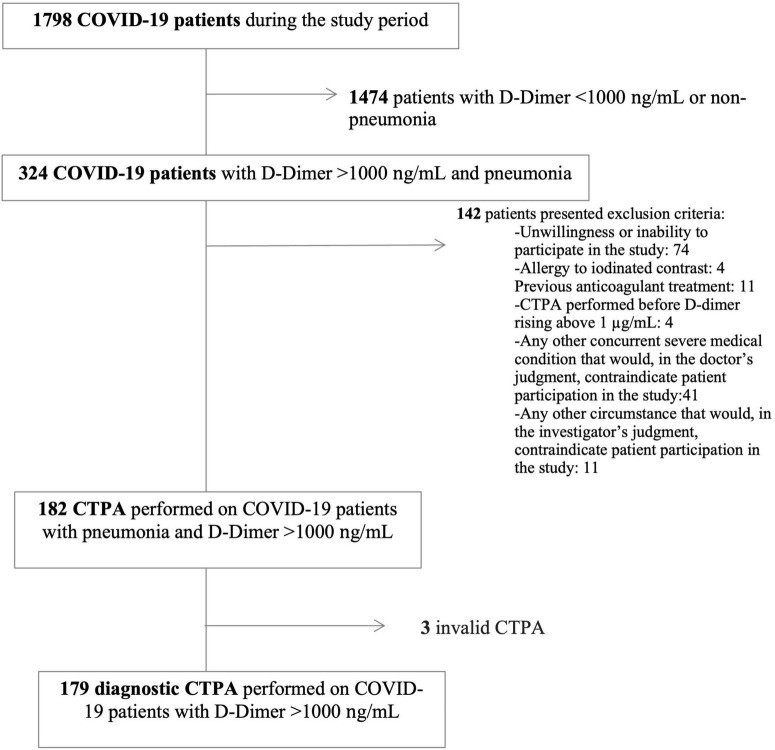
Flow chart. CTPA, computed tomography pulmonary angiography; PE, pulmonary embolism.

A total of 1,798 patients with COVID-19 were hospitalized during the study period (6 April 2020 to 2 February 2021). Overall, 324 of those patients had pneumonia with D-dimer > 1,000 ng/mL during hospitalization, and 142 of them presented at least one exclusion criterion. Ultimately, CTPA was performed on 182 patients, three of whom were excluded due to invalid CTPA (Figure 1).

In total, 179 patients were included in the analysis. Anthropometric and clinical characteristics are described in Table 1. The median age was 64.5 (55–74) years, and 66.5% of patients were men. The median time from symptom onset to hospital admission was 8 (4–10) days. The median CURB-65 score was 1 in the emergency department. Totally, 61 patients (34.3%) were admitted to the ICU.

**TABLE 1:** Baseline anthropometric and clinical characteristics of patients admitted because of COVID-19 pneumonia (all patients) and according to outcome (with and without pulmonary embolism).

	All patients (*n* = 179)	Non-PE patients (*n* = 108)	PE patients (*n* = 71)	*P*-value
Age, yrs.	64 (55–74)	63 (54–74)	65 (55–73)	0.79
Sex, male, *n* (%)	119 (66.5)	67 (62.0)	52 (73.2)	0.12
Body mass index, Kg/m^2^	28.8 (25.8–31.6)	28.5 (25.1–31.7)	29.6 (26.3–31.6)	0.18
Cardiovascular disease, *n* (%)	28 (15.6)	12 (11.1)	16 (22.5)	**0.04**
Arrhythmia, *n* (%)	8 (4.5)	6 (5.6)	2 (2.8)	0.48
Chronic respiratory disease, *n* (%)	19 (10.6)	12 (11.1)	7 (9.9)	0.79
Previous antiplatelet treatment, *n* (%)	30 (16.8)	21 (15.4)	9 (12.7)	0.24
Time from symptom onset to hospital admission, days	8 (4–10)	8 (4–10)	7 (4–11)	0.52
Time from symptom onset to CTPA, days	15 (10–24)	15 (9–26)	15 (10–22)	0.79
Current or former smokers, *n* (%)	67 (37.4)	41 (38.0)	26 (36.6)	0.86
Smoking, Pack-year	0 (0–10)	0 (0–15)	0 (0–10)	0.90
**Symptoms**				
Cough, *n* (%)	132 (74.2)	78 (72.2)	54 (77.1)	0.46
Fever, *n* (%)	125 (69.8)	73 (67.6)	52 (73.2)	0.42
Dyspnea, *n* (%)	106 (59.2)	65 (60.2)	41 (57.7)	0.75
Hemoptysis, *n* (%)	1 (0.6)	0 (0.0)	1 (1.4)	0.40
Chest pain, *n* (%)	11 (6.1)	5 (4.6)	6 (8.5)	0.35
**Physical examination**				
Respiratory rate, breaths per min	22 (20–27)	24 (18–28)	22 (20–26)	0.92
Heart rate, beats per min	87 (75–102)	87 (75–99)	86 (74–104)	0.77
Systolic BP, mm Hg	126 (114–137)	125 (113–138)	126 (117–135)	0.62
Diastolic BP, mm Hg	70 (62–79)	70 (60–78)	72 (63–80)	0.36
Temperature, ^°^C	36.8 (36.1–37.6)	36.8 (36.1–37.6)	36.9 (36.1–37.6)	0.86
Lower limb edema, *n* (%)	171 (95.5)	103 (95.4)	68 (95.8)	1
CURB 65	1 (0–2)	1 (0–2)	1 (1–2)	0.54
**Strong-moderate PE risk factors**				
Heart failure, *n* (%)	3 (1.7)	0 (0)	3 (4.2)	0.06
Fracture of lower limb	1 (0.6)	1 (0.9)	0 (0.0)	1
Chronic respiratory failure, *n* (%)	2 (1.1)	1 (0.9)	1 (1.4)	1
Neoplasm, *n* (%)	13 (7.3)	9 (8.3)	4 (5.6)	0.50
Previous VTE, *n* (%)	1 (0.6)	0 (0.0)	1 (1.4)	0.40
Myocardial infarction (within previous 3 months), *n* (%)	1 (0.6)	0 (0)	1 (1.4)	0.22
One or more known risk factors for PE, *n* (%)	20 (11.2)	11 (10.2)	9 (12.7)	0.61
**Treatment in hospital**				
**Oxygen therapy**				
Maximum FiO_2_	1.0 (0.3–1.0)	0.5 (0.3–1.0)	1.0 (0.4–1.0)	**0.00**
HFNC, *n* (%)	43 (24.2)	24 (22.4)	19 (26.8)	0.51
NIV, *n* (%)	5 (2.8)	3 (2.8)	2 (2.8)	1
IMV, *n* (%)	61 (34.3)	36 (33.6)	25 (35.2)	0.83
**Pharmacological therapy**				
Azithromycin, *n* (%)	28 (15.6)	18 (16.7)	10 (14.1)	0.64
Hydroxychloroquine, *n* (%)	36 (20.1)	18 (16.7)	18 (16.7)	0.16
Remdesivir, *n* (%)	27 (15.2)	18 (16.8)	9 (12.7)	0.45
Tocilizumab, *n* (%)	40 (22.3)	20 (18.5)	20 (28.2)	0.13
Other biological therapy, *n* (%)	7 (3.9)	3 (2.8)	4 (5.6)	0.44
Systemic steroids, *n* (%)	160 (89.4)	96 (88.9)	64 (90.1)	0.79
Clinical outcomes	17 (56.7)	9 (60.0)	8 (53.3)	0.71
Acute respiratory failure, *n* (%)	126 (73.7)	74 (71.8)	52 (76.5)	0.50
Arrhythmia, *n* (%)	3 (1.7)	1 (0.9)	2 (2.8)	0.56
ICU admission, *n* (%)	74 (41.3)	40 (37.0)	34 (47.9)	0.15
Death, *n* (%)	10 (5.6)	4 (3.7)	6 (8.5)	0.20

*Values represent percentage or median (IQR) according to its distribution. CTPA, computed tomography pulmonary angiography; BP, blood pressure; PE, pulmonary embolism; VTE, Venous thromboembolism; IVF, in vitro fertilization; FiO_2_, fractional inspired oxygen; HFNC, High Flow Nasal Cannula; NIV, non-invasive ventilation; IMV, invasive mechanical ventilation; ICU, intensive care unit. Bold indicates statistically significant differences between the groups.*

Most patients were on thromboprophylaxis with enoxaparin (154 with 40 mg per day and 20 with at least 1 mg/kg daily) before CTPA. The remaining five subjects were not on thromboprophylaxis before CTPA.

### Pulmonary Embolism Incidence Among COVID-19 Patients With Pneumonia

The overall incidence of PE was 39.7% (71 patients) (CI 95%, 32.4–47%). There were more patients with previous cardiovascular disease in the PE group when compared with those without. No significant differences were found in PE incidence according to age, sex, obesity, ICU admission, or type of specific treatment (hydroxychloroquine, remdesivir, tocilizumab, or systemic steroids) (Supplementary Table 1).

Totally, 20 patients presented at least one strong-moderate risk factor for PE (9 in the PE group and 11 in the non-PE group). Even when excluded from the analysis, the incidence of PE in the remaining population was still 39%.

There was a median 19-day interval between time from hospital admission and CTPA in Group B (*n* = 58), which was significantly longer than in the 121 patients in Group A (1; CI: 0–7 days). Furthermore, significant clinical and relevant differences were found between groups (Supplementary Tables 2–4).

### Comparison Between Patients With Pulmonary Embolism and Non-pulmonary Embolism

Age, sex, anthropometric, and clinical characteristics, physical examination, PE risk factors, and treatment during hospitalization were not significantly different between PE and non-PE individuals (Table 1). Moreover, no differences were found in the time from admission to CTPA [6.5 (1–19) vs. 7 (0–13) days, respectively].

Patients with PE required higher fractional inspired oxygen (FiO_2_), but no differences were found in the number of subjects requiring invasive/non-invasive ventilatory support or oxygen by high flow nasal cannula (Table 1). In addition, pharmacological therapy was similar during hospitalization (Table 1).

In total, 10 patients died during hospitalization (4 non-PE vs. 6 PE, *p* = 0.20). The main cause of death was respiratory failure. The time from admission to death was 17 days (10–33), with no significant differences between the two groups. Eight patients were readmitted in the follow-up, 50% of them in the PE group. No differences in ICU admission, acute respiratory failure, or arrhythmia were found between both groups during hospitalization (Table 1).

### Radiological Findings

The total lung involvement of COVID-19 was 16.7% in all patients. No differences were detected between the two groups [PE 19.9% (4.6–35.2)]; non-PE 15.5% (4.1–31); *p* = 0.75). PE showed a predominantly peripheral distribution, affecting at least one lobar, segmental, or subsegmental artery to 31, 42.3, and 26.8%, respectively. The overall PAOI and RV/LV ratios were 10% (5–22.5) and 0.9 (0.9–1.0), respectively. No difference in RV/LV ratio was found between groups.

### Laboratory Findings

Baseline laboratory findings, inflammatory, and PE biomarkers are shown in Tables 2, 3. Patients with PE showed higher neutrophil count and urea values when compared to patients with non-PE (*p* < 0.05). However, there were no significant differences in baseline coagulation function or arterial blood gas tests.

**TABLE 2:** Baseline laboratory data.

	All patients (*n* = 179)	Non-PE patients (*n* = 108)	PE patients (*n* = 71)	*P*-value
**Blood count, baseline**				
Hemoglobin, g/dL	13.6 (12.3–14.9)	13.5 (12–14.9)	13.7 (12.7–15)	0.19
Leucocyte count, 10^3^/μL	8.0 (5.5–11.4)	7.6 (5.4–10.1)	8.6 (5.6–12.0)	0.08
Neutrophil counts,%	78.4 (70.8–84.8)	76.1 (69.4–84.4)	81.3 (74.8–85.1)	**0.04**
**Biochemical profile, baseline**				
Glucose, mg/dL	125 (105–156)	121 (105–146)	136 (111–167)	0.08
ALT, U/L	29 (17–54)	26 (17–64)	34 (21–48)	0.6
Urea, mg/dL	35 (26–51)	33 (24–47)	40 (31–54)	**0.04**
Creatinine, mg/dL	0.8 (0.7–1.1)	0.8 (0.7–1.1)	0.9 (0.8–1.2)	0.06
Sodium, mEq/L	137 (135–140)	138 (135.5–140)	137 (135–140)	0.43
Potassium, mEq/L	4.1 (3.7–4.6)	4.0 (3.7–4.6)	4.1 (3.8–4.6)	0.34
Procalcitonin, ng/mL	64.5 (60.7– 68.1)	64.8 (60.7– 67.7)	64.4 (61.0– 68.7)	0.94
Cholesterol, mg/dL	147 (119–172)	141 (121–166)	149 (116–183)	0.26
Triglyceride, mg/dL	135 (103–191)	128 (98–180)	151 (111–199)	0.12
**Coagulation function, baseline**				
PT,%	81 (69–88)	81.5 (72–89)	79 (66–86)	0.10
INR	1.1 (1.1–1.3)	1.1 (1.1–1.2)	1.1 (1.1–1.3)	0.28
Fibrinogen, mg/dL	872 (723– 1,050)	881 (719– 1,052)	864 (744–1,050)	0.94
**Arterial blood test, baseline**				
PaO_2_/FiO_2_ ratio	276 (220–324)	286 (233–324)	262 (214–324)	0.14
Ph	7.46 (7.43–7.49)	7.46 (7.43–7.50)	7.46 (7.44–7.49)	0.74
PaO_2_, mmHg	62.5 (54.0–74.5)	63.0 (54.0–72.0)	62.0 (53.0–76.0)	0.74
PaCO_2_, mmHg	32.0 (29.0–36.0)	33.0 (30.0–36.0)	31.0 (28.0–35.0)	0.24

*Values represent median (IQR). ALT, alanine aminotransferase; PT, prothrombin time; PaO_2_, partial pressure of arterial blood oxygen; FiO_2_, fractional inspired oxygen; PaCO_2_, partial pressure of arterial blood carbon dioxide. Bold indicates statistically significant differences between the groups.*

**TABLE 3:** Inflammatory profile and pulmonary embolism biomarkers of patients admitted because of COVID-19 pneumonia with and without pulmonary embolism.

	All patients (*n* = 179)	Non-PE patients (*n* = 108)	PE patients (*n* = 71)	*P*-value
**LDH**				
Baseline, U/L	357 (283–498)	359 (284–528)	351 (281–488)	0.85
Peak, U/L	451 (340–611)	455.5 (337–626)	435 (340–590)	0.72
Prior to CTPA, U/L	345 (277–439)	324 (270–432)	351 (288–439)	0.49
**CRP**				
Baseline, mg/dL	10.2 (4.4–19.2)	9.7 (4.4–19.2)	10.9 (4.3–20.8)	0.59
Peak, mg/dL	15 (7.6–24.6)	14 (6.8–22.8)	16.2 (8.5–26.5)	0.25
Prior to CTPA, mg/dL	5.2 (1.5–13.3)	4.4 (1.3–11.5)	5.8 (1.7–15.4)	0.23
**ESR**				
Baseline, mm/h	69 (45–89)	69 (45–89)	70 (45.5–90)	0.81
Peak, mm/h	77 (61–103)	77 (57–104)	76 (65–94)	0.98
Prior to CTPA, mm/h	59 (35–85)	59 (35–85)	58 (34–81)	0.91
**D-dimer**				
Baseline, ng/mL	848 (294–2,329)	689 (256–1,672)	1,039 (324–4,888)	**0.02**
Peak, ng/mL	2,857 (1,909–4,960)	2,420 (1,616–3,655)	3,398 (2,376–8,537)	**0.00**
Prior to CTPA, ng/mL	1,809 (901–3,199)	1,532 (779–2,560)	2,381 (1,495–4,774)	**0.00**
**Ferritin**				
Baseline, ng/mL	711 (349–1,226)	764 (339–1,453)	628 (366–1,046)	0.36
Peak, ng/mL	872 (723–1,050)	881 (710–1,052)	864 (744–1,049)	0.94
Prior to CTPA, ng/mL	720 (354–1,313)	752 (368–1,462)	643 (349–1,110)	0.28
**D-dimer-to-ferritin ratio**				
Baseline	1.2 (0.3–3.8)	1 (0.3–2.1)	1.8 (0.4–12.5)	**0.03**
Peak	4.9 (2–10.2)	3.7 (1.9–8.8)	5.4 (2.6–12.5)	**0.05**
Prior to CTPA	2.3 (0.9–7.1)	1.8 (0.8–5.1)	3.4 (1.7–10.6)	**0.03**
**D-dimer-to-LDH ratio**				
Baseline	1.9 (0.8–6.8)	1.6 (0.7–4.4)	2.8 (0.9–11.8)	**0.01**
Peak	7.6 (4.6–11.9)	6.7 (3.9–10.9)	8.3 (6.1–16.9)	**0.01**
Prior to CTPA	5 (2.6–8.9)	3.5 (2.1–7.5)	6.3 (3.9–10.7)	**0.01**
**D-dimer-to-CRP ratio**				
Baseline	97.6 (35–364)	82.3 (33.9–227.8)	133.6 (36.3–554.6)	0.09
Peak	1157.3 (404.9–3905.7)	1086.4 (404.9–3505.2)	1230.6 (390.3–4118.9)	0.95
Prior to CTPA	442.4 (127.6–1474.6)	407.8 (110.6–1310.2)	446.3 (170.1–1602.9)	0.49
**Platelet count**				
Baseline, 10^3^/μL	210 (169–297)	204.5 (171–282)	230 (164–303)	0.54
Peak, 10^3^/μL	386 (288–495)	397 (288–505)	351 (288–481)	0.22
Prior to CTPA, 10^3^/μL	279 (198–354)	274 (193.5–350.5)	284 (203–371)	0.52
**Lymphocyte counts**				
Baseline,%	13.5 (8.5–19.7)	14.3 (9.4–20.5)	11 (7.7–15.9)	**0.03**
Peak*,%	7 (4.5–12.2)	8.2 (4.7–13.6)	5.8 (3.7–9)	**0.03**
Prior to CTPA,%	15.9 (10.2–25.9)	18.3 (11.8–28.8)	13.2 (7.9–19.8)	**0.00**
**NLR**				
Baseline	5.8 (3.7–10.1)	5.2 (3.4–8.8)	7.2 (4.6–10.7)	**0.02**
Peak	12.4 (6.8–20.1)	10.6 (5.8–19)	15 (9.7–24.9)	**0.02**
Prior to CTPA	4.7 (2.5–8.1)	4.1 (2.2–6.9)	5.5 (3.5–10.2)	**0.00**
**PDW,%**				
Baseline	16.3 (15.8–16.8)	16.1 (15.7–16.8)	16.6 (16.1–17.2)	**0.00**
Peak	17.2 (16.9–17.9)	17.1 (16.8–17.7)	17.3 (16.9–18.2)	**0.04**
Prior to CTPA	16.4 (15.9–16.8)	16.2 (15.8–16.7)	16.6 (16.1–16.9)	**0.01**
IL-6, pg/mL peak	62.5 (23–181)	60.4 (19.6–19.5)	64 (31–140)	0.77
IL-10, pg/mL peak	8 (4.3–13.8)	9.2 (5.8–18.5)	5.1 (3.5–10.9)	**0.01**
NT-pro BNP, pg/mL peak	234 (100–605)	215 (93–532)	248 (127–701)	0.35
hs Troponin I, ng/L peak	9.8 (4.1–28.3)	8.9 (3.7–25.3)	10.9 (4.2– 34.9)	0.44
Fibrinogen, mg/dL peak	872 (723–1,050)	881 (710–1,052)	864 (744–1,049)	0.94

Values represent median (IQR). Baseline, first variable value; Peak, maximum value; Peak*, minimum value; Prior to CTPA, previous value to CTPA. CTPA, computed tomography pulmonary angiography; LDH, lactate dehydrogenase; CRP, C-reactive protein; ESR, erythrocyte sedimentation rate; IL-6, interleukin-6; NLR, neutrophil-to-lymphocyte ratio; NT-proBNP, N-terminal pro hormone B-type natriuretic peptide; PDW, platelet distribution width. Bold indicates statistically significant differences between the groups.

Higher baseline, peak and prior-to-CTPA D-Dimer, D-dimer-to-ferritin and D-dimer-to-LDH ratios, platelet distribution width (PDW), and neutrophil-to-lymphocyte ratio (NLR) values were found in patients with PE when compared to patients with non-PE (*p* < 0.05 all variables). Moreover, baseline, minimum, and prior-to-CTPA lymphocyte counts were found to be lower in patients with PE, while no differences were detected in platelet count, ESR, CRP, LDH, ferritin, IL-6, NT-proBNP, troponin, or fibrinogen values. In contrast, IL-10 concentration was lower in patients with PE (Table 3).

In patients with PE, bivariate analysis revealed significant correlations between the PAOI and baseline (rho = 0.24, *p* = 0.045), prior to CTPA (rho = 0.25, *p* = 0.04), peak (rho = 0.27, *p* = 0.02) D-dimer values, D-dimer-to-LDH ratio (rho = 0.28, *p* = 0.04) on admission, and peak IL-10 levels (rho = −0.31, *p* = 0.03).

### Pulmonary Embolism Predictive Score

**FIGURE 2:**
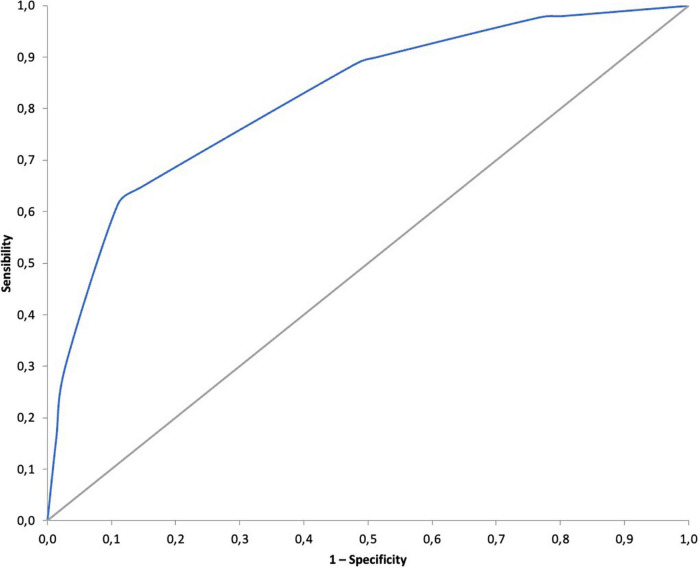
Area under the ROC curve (AUC) of the PATCOM score. PATCOM, Pulmonary Artery Thrombosis in COVID-19 Mallorca; ROC, receiver operating characteristic.

A total of 11 variables related to the presence of PE in the regression analysis were dichotomized in order to construct a PE predictive score with Group A patients. After dichotomization, multivariable logistic regression analysis led to a selection of four variables (Supplementary Table 5). Table 4 shows the constructed prediction score with the calculated weight and cut-off points of the variables. The PATCOM score stands for *Pulmonary Artery Thrombosis in COVID-19 Mallorca* and included the platelet count, PDW, urea concentration, and D-dimer-to-ferritin ratio. The AUC-ROC of the PATCOM score was 0.81 (95% CI: 0.73–0.89). The suggested score ranged from 0 to 4 points. The probability of PE during the hospitalization was low (5.6%) at 0 points, moderate (25%) at 1–2 points, and high (77.1%) at 3–4 points (Figure 2). A value ≥ 3 was predictive of PE with a sensitivity of 61% and a specificity of 89%. Supplementary Table 6 summarizes sensitivity, specificity, and positive and negative predictive values of different cut-off points. The model explained 39.1% (Nagelkerke *R*^2^) of the variance. HL was 0.942, confirming an appropriate goodness-of-fit.

**TABLE 4:** The PATCOM score.

	Value
**Platelet count**	
<280 10^3^/μL	0
≥280 10^3^/μL	1
**PDW**	
<16%	0
≥16%	1
**Urea**	
<35 mg/dL	0
≥35 mg/dL	1
**D-dimer-to-ferritin ratio**	
<3	0
≥3	1

*PATCOM, Pulmonary Artery Thrombosis in COVID-19 Mallorca.*

## Discussion

To the best of our knowledge, this is the largest prospective study to date systematically exploring the real incidence of PE in a cohort of patients with COVID-19 admitted to a hospital. Additionally, taking several variables together, our study enabled us to develop a PE score that could be used as a prediction rule for this population, once adequately validated.

### Previous Studies

The increased risk of PE in COVID-19 has been described previously, yet with a high variability of reported rates (7, 8, 22–24) and contradictory data (15, 16). The true incidence of PE is not clear, since diagnostic tests were mostly performed only when thrombotic complications were clinically suspected, which is challenging, as symptoms of moderate-severe COVID-19 overlap with PE. In addition, all these studies were retrospective (7, 8, 15, 16, 21–25). We present the largest prospective study to date evaluating the incidence of PE. We found a very high incidence of PE (up to 39.7%) in consecutive hospitalized COVID-19 patients with pneumonia and increased D-dimer, regardless of clinical suspicion, which is consistent with data reported in two previous retrospective studies. In the first one, CTPA was performed in all patients with increased D-dimer (>500 ng/mL), whereas in the second one, it was performed in all consecutive ICU patients (10, 13). As far as we know, only two prospective studies have systematically explored the incidence of PE in all consecutive patients with COVID-19. García-Ortega et al. included all patients from a single hospital when D-dimer was increased (> 500 ng/mL or > age × 10 in patients > 50 years), and the frequency of PE was also high 35.6% (95% CI 29.6–41.6) despite thromboprophylaxis (11). However, this study is of limited applicability because of its small sample size (*n* = 73). In contrast, Jevnikar et al. found a lower incidence of PE (14%) in 106 consecutive patients with COVID-19, showing the incidence in all patients at the time of hospital admission regardless of COVID-19 severity or D-dimer value (9). In addition, there was a 15-day median interval between symptom onset and CTPA in our study, which is longer than the 7 days in the latter study. Remarkably, recent data found that the incidence of PE was increased approximately 2–3-fold during the first 2 weeks in hospitalized patients with COVID-19, supporting the hypothesis of a delayed procoagulant state throughout the second phase of the disease following the cytokine storm (30).

### D-Dimer and Other Potential Biomarkers of Pulmonary Embolism in COVID-19

Relationships between COVID-19 and PE are very complex, with many factors and confounding variables yet to be clarified. D-dimer, a degradation product of cross-linked fibrin, is considered to reflect the global activity of clot formation and lysis, and increased levels have been correlated with COVID-19 severity (2–5). In addition, D-dimer levels have been found to be higher in COVID-19 patients with PE (7, 9–11, 22, 23). However, confusion regarding the type of DDUs, either as DDU or fibrinogen equivalent units (FEU), can create incorrect conclusions, as the FEU value is two times that of DDU, and some of the abovementioned studies did not report any DDU (9, 10, 13, 31). Further studies should harmonize D-dimer levels so as to ensure the comparability of different assays (32). In the present study, all the patients included presented D-dimer > 1,000 ng/mL, but patients with PE showed even higher values than non-PE subjects. Further, it is worth noting that, despite inflammation biomarkers being equally high in both groups, there were significant differences in D-dimer-to-ferritin and D-dimer-to-LDH ratios, with both proving to be higher in the PE group. Moreover, other biomarkers, such as PDW and NLR, were significantly higher in patients with PE as compared to patients without PE. Interestingly, both PDW and NRL have also been described as severity markers in COVID-19 (33, 34), with more increased NLR among PE cases (35). Higher PDW, a surrogate marker of platelet activation, has also been found in non-COVID-19 PE patients (36). This whole scenario with higher D-dimer, NLR, and PDW values in patients with PE, together with the fact that deep vein thrombosis (DVT) is uncommon in hospitalized COVID-19 individuals (37, 38), supports the hypothesis that SARS-CoV-2 could induce direct alveolar damage, promoting a local immunothrombosis phenomenon in the pulmonary arteries.

### Clinical Implications

Retrospective data showed that PE is associated with both increased mortality and length of hospital stay in patients with COVID-19 (13, 23). By contrast, we found no significant differences in clinical outcomes when comparing patients with PE to patients with non-PE, which may be due to a notable influence of early detection and a prompt start of anticoagulant treatment (39). However, since thrombi were mainly peripheral with a low overall thrombotic load, they could have had a minor influence on clinical outcomes. Our results, however, are in accordance with other prospective studies (9, 10). Nonetheless, the sample size of the present study may not be sufficiently powered to find differences in these objectives.

In our study, as in most of the abovementioned ones, PE incidence was high despite much of the population being on anticoagulant thromboprophylaxis. Recent randomized trials showed that full anticoagulation with heparin may be clinically favorable in COVID-19 (39, 40), raising uncertainties about the selection of higher risk patients, appropriate evaluations, and interventions to prevent and/or treat PE. General scores, such as Wells and revised Geneva (18), used as PE prediction rules have also been evaluated in COVID-19 (most of them assessed in retrospective populations), but results have been heterogeneous and mainly poor (41).

To our knowledge, there is only one specific score constructed including 73 hospitalized patients with COVID-19 that showed excellent accuracy (AUC-ROC of 0.86; CI: 0.80–0.93) (11). We also developed a new specific rule (PATCOM score) in COVID-19 patients with pneumonia with a remarkable AUC-ROC of 0.81 (95% CI: 0.73–0.89) in 121 patients who either did not require ICU or had CTPA performed before ICU admission. Values greater than 2 identified patients with a very high probability of PE (77.1%), and consequently, needed a CTPA to be performed promptly, along with anticoagulation at full doses. Interestingly, when the PATCOM score was 0, a low incidence of PE (5.6%) was detected. However, the variables of these scores were dissimilar, maybe due to differences in inclusion criteria, and the populations and biomarkers were evaluated. Nevertheless, although both scores are promising, internal and external validations are warranted before their use in clinical settings.

### Strengths and Limitations

The present study has several strengths, such as its prospective enrollment, with a previously calculated sample size, clear inclusion/exclusion criteria, and intensive characterization from the point of view of clinical, imaging, and complete laboratory examinations point of view, including inflammatory and coagulation biomarkers. Additionally, this is the largest prospective study demonstrating a high incidence of PE in patients with COVID-19, regardless of clinical suspicion, and representing the whole spectrum of hospitalized COVID-19 (ICU and non-ICU). Nonetheless, as in any study, there are some potential limitations that deserve some consideration. First, lower limb duplex ultrasound was not routinely performed; therefore, asymptomatic events could not be ruled out; although, most previous studies found a relatively low incidence of DVT in COVID-19 (37, 38). Second, this study was carried out before the Omicron variant had become the dominant form of SARS-CoV-2 and with low vaccination rates (none of the patients were vaccinated against SARS-CoV-2), all of which may have had an impact on the incidence of PE. Finally, CTPA was routinely performed in patients with D-dimer > 1,000 ng/mL; therefore, the usefulness of the PATCOM score and the incidence of PE among the remaining patients with SARS-CoV-2 pneumonia are unknown.

## Conclusion

In conclusion, we have demonstrated a very high incidence (39.7%) of PE in consecutively admitted COVID-19 patients with pneumonia and D-dimer values > 1,000 ng/mL, regardless of clinical suspicion. Further, patients with PE had higher D-dimer levels both in absolute terms and relative to systemic inflammation biomarkers and significant differences in PDW, NLR, and lymphocyte count. In addition, patients with PE required higher O_2_ concentrations, although no differences in clinical outcomes were found. Our findings could be relevant to the management of these patients and could help in clinical decision-making, such as performing CTPA or starting empirical anticoagulation. We have developed the 4-variable PATCOM score as a promising easy prediction PE rule, which needs to be further validated.

## Data Availability Statement

Due to the applicable privacy regulation and Good Clinical Practices legislation, the full underlying dataset supporting the study cannot be provided. This dataset contains potentially identifying information, for example, age, BMI, and data of admission to the hospital leading to a unique subject in the dataset. Moreover, sharing individual participant data with third parties was not specifically included in the informed consent form of the study, and unrestricted diffusion of such data may pose a potential threat of revealing participants’ identities, as permanent data anonymization was not carried out. However, other researchers who meet the criteria for access to confidential data may request to gain access to the minimal data set underlying the results under request at the Ethics Committee (contact via: https://www.caib.es/sites/comiteetic/es/portada44578/?campa=yes) (e-mail address: ceic_ib@caib.es). Besides, the researchers shall submit a methodological proposal.

## Ethics Statement

The Institutional Ethics Committee of the Balearic Islands approved the study (IB 4197/20 PI), and all subjects gave their written informed consent. Only patients with a critical clinical condition gave verbal consent instead, in front of at least two witnesses.

## Author Contributions

CS, AA-F, ES-L, NT-P, and JS conceptualized, administered, and supervised the study. AA-F designed the study protocol with the help of NT-P. AA-F analyzed the data. AA-F and BN were responsibility for funding acquisition. NC, LR-C, JM, SH, DM-G, JB, and LM contributed to patient recruitment and data collection. AA-F attested that all listed authors meet authorship criteria. All authors were involved in further drafts of the manuscript and revised it critically for content and approved the final version of the manuscript.

## Conflict of Interest

AA-F reports personal fees from Astrazeneca, GSK, Chiesi, and Menarini, from outside the submitted work. JS reports personal fees, as a speaker, from Roche and Boehringer-Ingelheim, with no relationship with the submitted work. ES-L reports personal fees from Astrazeneca, GSK, and Boehringer-Ingelheim, personal and advisory board fees from MSD, and grants, personal, and advisory board fees from Actelion and Janssen, from outside the submitted work. BN reports personal fees from Roche, Brystol, and Boehringer-Ingelheim, with no relationship with the submitted work. The remaining authors declare that the research was conducted in the absence of any commercial or financial relationships that could be construed as a potential conflict of interest.

## Publisher’s Note

All claims expressed in this article are solely those of the authors and do not necessarily represent those of their affiliated organizations, or those of the publisher, the editors and the reviewers. Any product that may be evaluated in this article, or claim that may be made by its manufacturer, is not guaranteed or endorsed by the publisher.
