# Supplementary material for: A Prospective Study Evaluating Cumulative Incidence and a Specific Prediction Rule in Pulmonary Embolism in COVID-19
Source: Front Med (Lausanne). 2022 Jul 1;9:936816. doi: 10.3389/fmed.2022.936816 (PMC9283900; doi:10.3389/fmed.2022.936816)
Supplement: Supplementary file 1 [file Data_Sheet_1.docx]

**Supplemental Table E1. Title: Differences in incidence among groups**

|  | **All patients**  **(n= 179)** | **PE patients**  **(n= 71)** | **Non-PE**  **patients**  **(n=108)** | **PE**  **incidence** | **p value** |
| --- | --- | --- | --- | --- | --- |
| **Sex** |  |  |  |  | 0.12 |
| **Men, n (%)** | 119 (66.5) | 52 (73.2) | 67 (62) | 52 (43.7) |  |
| **Women, n (%)** | 60 (33.5) | 19 (26.8) | 41 (38.0) | 19 (31.7) |  |
| **Age** |  |  |  |  | 0.74 |
| **≥65, years, n (%)** | 88 (49.2) | 36 (50.7) | 52 (48.1) | 36 (40.9) |  |
| **<65, years, n (%)** | 91 (50.8) | 35 (49.3) | 56 (51.9) | 35 (38.5) |  |
| **Obesity** |  |  |  |  | 0.25 |
| **BMI≥30, n (%)** | 59 (35.8) | 26 (41.3) | 33 (32.4) | 26 (44.1) |  |
| **BMI<30, n (%)** | 106 (64.2) | 37 (58.7) | 69 (67.6) | 37 (34.9) |  |
| **ICU admission** |  |  |  |  | 0.15 |
| **Yes, n (%)** | 74 (41.3) | 34 (47.9) | 40 (37.0) | 34 (45.9) |  |
| **No, n (%)** | 105 (58.7) | 37 (52.1) | 68 (63.0) | 37 (35.2) |  |
| **Hydroxychloroquine** |  |  |  |  | 0.16 |
| **Yes, n (%)** | 36 (20.1) | 18 (25.4) | 18 (16.7) | 18 (50.0) |  |
| **No, n (%)** | 143 (79.9) | 53 (74.6) | 90 (83.3) | 53 (37.1) |  |
| **Remdesivir** |  |  |  |  | 0.45 |
| **Yes, n (%)** | 27 (15.2) | 9 (12.7) | 18 (16.8) | 9 (33.3) |  |
| **No, n (%)** | 151 (84.8) | 62 (87.3) | 89 (83.2) | 62 (41.1) |  |
| **Tocilizumab** |  |  |  |  | 0.13 |
| **Yes, n (%)** | 40 (22.3) | 20 (28.2) | 20 (18.5) | 20 (50.0) |  |
| **No, n (%)** | 139 (77.7) | 51 (71.8) | 88 (81.5) | 51 (36.7) |  |
| **Systemic steroids** |  |  |  |  | 0.79 |
| **Yes, n (%)** | 160 (89.4) | 64 (90.1) | 96 (88.9) | 64 (40.0) |  |
| **No, n (%)** | 19 (10.6) | 7 (9.9) | 12 (11.1) | 7 (36.8) |  |

Legend: Abbreviations: BMI, body mass index

**Supplemental Table E2. Title: Baseline anthropometric and clinical characteristics of patients who either did not require ICU-level care or underwent a CTPA before their ICU admission (A), and ICU patients that underwent CTPA during or after ICU admission (B)**

|  | **A**  **(n=121)** | **B**  **(n= 58)** | **p value** |
| --- | --- | --- | --- |
| **Age, yrs.** | 66 (57-76) | 59 (53-66) | **0.03** |
| **Sex, male, n (%)** | 79 (65.3) | 40 (69.0) | 0.62 |
| **Body mass index, Kg/m^2^** | 28.3 (25.5-31.1) | 29.7 (27.2-31.9) | 0.13 |
| **Cardiovascular disease, n (%)** | 20 (16.5) | 8 (13.8) | 0.63 |
| **Arrhythmia, n (%)** | 6 (5) | 2 (3.4) | 1.00 |
| **Chronic kidney disease, n (%)** | 13 (10.7) | 1 (1.7) | **0.03** |
| **Chronic respiratory disease, n (%)** | 17 (14.0) | 2 (3.4) | **0.03** |
| **Hypertension, n (%)** | 64 (52.9) | 19 (32.8) | **0.01** |
| **NSAIDs, n (%)** | 19 (15.7) | 3 (5.2) | **0.04** |
| **Previous antiplatelet treatment, n (%)** | 23 (19) | 7 (12.1) | 0.25 |
| **Time from symptom onset to hospital admission, days** | 7 (4-10) | 8 (6-10) | 0.52 |
| **Time from symptom onset to CTPA, days** | 11 (7-16) | 27 (20-41) | **0.00** |
| **Time from symptom onset to hospital discharge or death, days** | 13 (9-19) | 30 (21-50) | **0.00** |
| **Current or former smokers, n (%)** | 46 (38) | 21 (36.2) | 0.87 |
| **Total lung infection, (%)** | 9.4 (3.3-29.6) | 25.9 (15.9-43.2) | **0.00** |
| **Symptoms** |  |  |  |
| **Cough, n (%)** | 91 (75.2) | 41 (75.9) | 0.64 |
| **Fever, n (%)** | 82 (67.8) | 43 (74.1) | 0.38 |
| **Dyspnea, n (%)** | 68 (56.2) | 38 (56.5) | 0.23 |
| **Hemoptysis, n (%)** | 1 (8) | 0 (0) | 1.00 |
| **Chest pain, n (%)** | 6 (5) | 5 (8.6) | 0.33 |
| **Physical examination*** |  |  |  |
| **Respiratory rate, breaths per min** | 22 (18-25) | 24.5 (20-32) | **0.03** |
| **Heart rate, beats per min** | 87 (75-102) | 85.5 (75-102) | 0.71 |
| **Systolic BP, mm Hg** | 125 (114-135) | 126 (113-139) | 0.84 |
| **Diastolic BP, mm Hg** | 70 (62-78) | 71 (64-80) | 0.63 |
| **Temperature, ºC** | 36.8 (36.1-37.5) | 37 (36.1-37.8) | 0.40 |
| **Lower limb edema, n (%)** | 3 (2.5) | 1 (1.7) | 1 |
| **CURB 65** | 1 (0-2) | 1 (1-2) | 0.51 |
| **Strong-moderate PE risk factors** |  |  |  |
| **Heart failure, n (%)** | 2 (1.7) | 1 (1.7) | 1 |
| **Fracture of lower limb** | 1 (0.8) | 0 (0.0) | 1 |
| **Chronic respiratory failure, n (%)** | 2 (1.7) | 0 (0) | 0.47 |
| **Neoplasm, n (%)** | 10 (8.3) | 3 (5.2) | 0.55 |
| **Previous VTE, n (%)** | 1 (0.8) | 0 (0) | 1 |
| **Myocardial infarction (within previous 3 months), n (%)** | 0 (0) | 1 (1.7) | 0.32 |
| **One or more known risk factors for PE, n (%)** | 15 (12.4) | 5 (8.6) | 0.45 |
| **Treatment in hospital** |  |  |  |
| **Oxygen therapy** |  |  |  |
| **Maximum FiO_2_** | 1 (0.35-1) | 1.0 (1-1) | **0.00** |
| **HFNC, n (%)** | 21 (17.4) | 22 (38.6) | **0.00** |
| **NIV, n (%)** | 1 (8) | 4 (6.9) | **0.03** |
| **IMV, n (%)** | 12 (10) | 49 (84.5) | **0.00** |
| **Pharmacological therapy** |  |  |  |
| **Azithromycin, n (%)** | 0 (0) | 0 (0) | **0.03** |
| **Hydroxychloroquine, n (%)** | 0 (0) | 0 (0) | 0.18 |
| **Remdesivir, n (%)** | 18 (15) | 9 (15.5) | 0.92 |
| **Tocilizumab, n (%)** | 19 (15.7) | 21 (36.2) | **0.02** |
| **Other biological therapy, n (%)** | 3 (2.5) | 4 (7) | 0.21 |
| **Antibiotic treatment, n (%)** | 64 (53) | 47 (81) | **0.00** |
| **Bolus administration of steroid therapy, n (%)** | 105 (86.8) | 55 (94.8) | 0.1 |
| **Clinical outcomes** | 9 (60.0) | 8 (53.3) | 0.71 |
| **Acute respiratory failure, n (%)** | 53 (46.5) | 45 (80.4) | **0.00** |
| **Arrhythmia, n (%)** | 1 (8) | 3 (2.4) | 0.24 |
| **ICU admission, n (%)** | 16 (13.2) | 58 (100) | **0.00** |
| **Death, n (%)** | 6 (5.0) | 4 (6.9) | 0.73 |

Legend: Values represent percentage or median (IQR) according to its distribution. Abbreviations: CTPA, computed tomography pulmonary angiography; BP, blood pressure; PE, pulmonary embolism; VTE, Venous thromboembolism; IVF, in vitro fertilization; FiO2, fractional inspired oxygen; HFNC, High Flow Nasal Cannula; NIV, non-invasive ventilation; NSAIDs, Non-steroidal anti-inflammatory drugs; IMV, invasive mechanical ventilation; ICU, intensive care unit

**Supplemental Table E3. Title: Baseline laboratory data of patients who either did not require ICU-level care or underwent a CTPA before their ICU admission (A), and ICU patients that underwent imaging diagnostics during or after ICU admission (B)**

|  | **A**  **(n=121)** | **B**  **(n= 58)** | **p value** |
| --- | --- | --- | --- |
| **Blood count, baseline** |  |  |  |
| **Hemoglobin, g/dL** | 13.6 (12.1-14.7) | 13.9 (12.9-15.3) | 0.06 |
| **Leucocyte count, 10^3^/µL** | 7.8 (5.3-11.4) | 8.3 (6.2-10.3) | 0.24 |
| **Neutrophil counts, %** | 76.3 (68.7-83.9) | 83 (74.8-85.8) | **0.00** |
| **Biochemical profile, baseline** | |  |  |
| **Glucose, mg/dL** | 124 (104-152) | 134 (110-173) | 0.18 |
| **Urea, mg/dL** | 35 (24-53) | 36.5 (29-48) | 0.96 |
| **Creatinine, mg/dL** | 0.8 (0.7-1.1) | 0.8 (0.7-1) | 0.06 |
| **Sodium, mEq/L** | 138 (136-140) | 137 (134-140) | 0.14 |
| **Potassium, mEq/L** | 4.1 (3.7-4.6) | 4.0 (3.7-4.2) | **0.02** |
| **Procalcitonin, ng/mL** | 0.17 (0.07-0.47) | 0.16 (0.09-0.42) | 0.53 |
| **Albumin, g/L** | 34.3 (31.5-37) | 33.1 (29.8-35.5) | **0.07** |
| **Cholesterol, mg/dL** | 141.5 (120.5-161) | 156 (118-186.5) | 0.21 |
| **Triglyceride, mg/dL** | 123.5 (96-168) | 164.5 (120-272) | **0.00** |
| **Coagulation function, baseline** | |  |  |
| **PT,s** | 13 (12-14.4) | 12.5 (12-13.4) | 0.22 |
| **PT, %** | 78 (68-88) | 82.5 (71-87) | 0.38 |
| **Fibrinogen, mg/dL** | 722 (579-876) | 616 (431-763) | **0.00** |

Legend: Values represent median (IQR). Abbreviations: ALT, alanine aminotransferase; PT, prothrombin time; PaO2, partial pressure of arterial blood oxygen; FiO2, fractional inspired oxygen; PaCO2, partial pressure of arterial blood carbon dioxide

**Supplemental Table E4. Title: Inflammatory profile and pulmonary embolism biomarkers of patients who either did not require ICU-level care or underwent a CTPA before their ICU admission (A), and ICU patients that underwent imaging diagnostics during or after ICU admission (B)**

|  | **A**  **(n=121)** | **B**  **(n= 58)** | **p value** |
| --- | --- | --- | --- |
| **LDH** |  |  |  |
| **Baseline, U/L** | 336 (267-441) | 452 (310-614) | **0.00** |
| **Peak, U/L** | 386 (300.5-527) | 595 (475-682) | **0.00** |
| **Prior to CTPA, U/L** | 342 (269.5-435.5) | 352 (292-426) | 0.51 |
| **CRP** |  |  |  |
| **Baseline, mg/dL** | 10 (4.5-16.9) | 13.4 (3.1-22.6) | 0.42 |
| **Peak, mg/dL** | 12.4 (6.8-21.6) | 17.9 (10.8-28.7) | **0.00** |
| **Prior to CTPA, mg/dL** | 5.9 (1.7-13.4 | 2.7 (0.8-9.9) | **0.05** |
| **ESR** |  |  |  |
| **Baseline, mm/h** | 71 (46-91) | 68 (41.5-87.5) | 0.35 |
| **Peak, mm/h** | 77 (57-104) | 76 (64-101) | 0.87 |
| **Prior to CTPA, mm/h** | 62 (36-85) | 50 (27-69) | 0.14 |
| **D-dimer** |  |  |  |
| **Baseline, µg/mL** | 1205 (350-3149) | 418 (227-699) | **0.00** |
| **Peak, µg/mL** | 2845 (1930-5216) | 2924 (1785-4038) | 0.59 |
| **Prior to CTPA, µg/mL** | 2142 (1394-3585) | 887.5 (505-1903) | **0.00** |
| **Ferritin** |  |  |  |
| **Baseline, ng/mL** | 611 (313-1071) | 859 (535-1873) | 0.10 |
| **Peak, ng/mL** | 799 (391-1671) | 1926 (997-3235) | **0.00** |
| **Prior to CTPA, ng/mL** | 601 (283-1079) | 916 (491-1525) | 0.08 |
| **D-dimer-to-ferritin ratio** |  |  |  |
| **Baseline** | 1.7 (0.6-7.8) | 0.7 (0.2-1.3) | **0.00** |
| **Peak** | 5.7 (2.2-12.5) | 3.4 (1.6-5.9) | **0.00** |
| **Prior to CTPA** | 3.3 (1.5-11) | 1.2 (0.6-3) | **0.00** |
| **D-dimer-to-LDH ratio** |  |  |  |
| **Baseline** | 3 (1-8.9) | 0.8 (0.5-1.6) | **0.00** |
| **Peak** | 8 (5-13) | 6.6 (3.9-9.9) | **0.04** |
| **Prior to CTPA** | 3.5 (2.1-7.5) | 6.3 (3.4-10.5) | **0.00** |
| **D-dimer-to-CRP ratio** |  |  |  |
| **Baseline** | 127 (50-484) | 35(19-133) | **0.00** |
| **Peak** | 1251(478-4266) | 916 (228-3200) | 0.19 |
| **Prior to CTPA** | 407 (170-1474) | 449 (112-1461) | 0.69 |
| **Platelet count** |  |  |  |
| **Baseline, 10^3^/µL** | 210 (174-295) | 213 (164-297) | 0.83 |
| **Peak, 10^3^/µL** | 350 (280-474) | 417 (338-503) | **0.00** |
| **Prior to CTPA, 10^3^/µL** | 263 (183-332) | 292 (228-385) | **0.02** |
| **Lymphocyte count** |  |  |  |
| **Baseline, %** | 15.1 (8.7-20.6) | 10.8 (7.4-15.2) | **0.02** |
| **Peak*, %** | 9.3 (5.6-15.6) | 4.6 (3.1-6.1) | **0.00** |
| **Prior to CTPA, %** | 14.3 (9.9-23.9) | 17.5 (13.4-29.4) | **0.03** |
| **NLR** |  |  |  |
| **Baseline** | 5 (3.3-9.3) | 7.7 (4.9-11.3) | **0.00** |
| **Peak** | 9 (5-15.7) | 19 (14.6-129.6) | **0.00** |
| **Prior to CTPA** | 5 (2.7-8.5) | 4 (1.9-6.1) | **0.02** |
| **PDW, %** |  |  |  |
| **Baseline** | 16.4 (15.8-16.7) | 16.3 (16.0-16.7) | 0.94 |
| **Peak** | 17.1 (16.7-17.5) | 17.6 (17.1-18.3) | **0.00** |
| **Prior to CTPA** | 16.4 (15.9-16.9) | 16.3 (15.9-16.7) | 0.27 |
| **IL-6, pg/mL peak** | 70 (27-175) | 59 (15-214) | 0.47 |
| **IL-10, pg/mL peak** | 8.1 (4.3-10.1) | 7 (4.2-14.5) | 0.86 |
| **NT-pro BNP, pg/mL peak** | 248 (104-573) | 216 (98-630) | 0.88 |
| **hs Troponin I, ng/L peak** | 8.3 (3,6-35) | 11.1 (4.3-28) | 0.67 |
| **Fibrinogen, mg/dL peak** | 794 (683-974) | 1023 (879-1129) | **0.00** |

Legend: Values represent median (IQR). Baseline, first variable value; Peak, maximum value; Peak*, minimum value; Prior to CTPA, previous value to CTPA. Abbreviations: CTPA, computed tomography pulmonary angiography; LDH, lactate dehydrogenase; CRP, C-reactive protein; ESR, erythrocyte sedimentation rate; IL-6, interleukin-6; NLR, neutrophil-to-lymphocyte ratio; NT-proBNP, N-terminal pro hormone B-type natriuretic peptide; PDW, platelet distribution width

**Supplemental Table E5. Title: Regression analysis**

|  | **Simple linear regression** | | | **Multivariable linear regression** | | |
| --- | --- | --- | --- | --- | --- | --- |
| **Variable** | **Crude**  **Odds ratio (CI)** | **p value** | | **Adjusted**  **Odds ratio (CI)** | **p value** | |
| **Age** | 1.01 (0.99-1.04) | | 0.29 |  | |  |
| **BMI** | 1.04 (0.98-1.10) | | 0.25 |  | |  |
| **Cardiovascular disease** | 3.66 (1.34-10.02) | | **0.01** |  | |  |
| **Baseline neutrophils ≥ 75%** | 3.62 (1.63-8.06) | | **0.00** |  | |  |
| **Baseline platelets ≥ 280 10^3^/µL** | 3.18 (1.42-7.12) | | **0.01** | 2.88 (1.11-7.51) | | **0.03** |
| **Baseline plaquetocrit ≥ 0.24%** | 2.96 (1.23-7.13) | | **0.02** |  | |  |
| **Baseline PDW ≥ 16** | 3.40 (1.44-8.02) | | **0.01** | 3.34 (1.18-9.43) | | **0.02** |
| **Baseline NLR ≥ 4** | 4.37 (1.80-10.62) | | **0.00** |  | |  |
| **Baseline urea ≥ 35 mg/dL** | 4.20 (1.90-9.30) | | **0.00** | 3.90 (1.52-10.02) | | **0.00** |
| **Baseline creatinine ≥ 0.84 mg/dL** | 3.08 (1.40-6.75) | | **0.00** |  | |  |
| **Baseline D-dimer-to-ferritin ratio ≥ 3** | 6.23 (2.73-14.26) | | **0.00** | 4.83 (1.96-11.92) | | **0.00** |

Legend: Abbreviations: BMI, body mass index; NLR, neutrophil-to-lymphocyte ratio; PDW, platelet distribution width

**Supplemental Table E6. Title: Sensitivity, specificity, PPV and NPV of different PATCOM score cut-off points for PE diagnosis in COVID-19 pneumonia patients**

|  | **PATCOM ≥ 1** | **PATCOM ≥ 2** | **PATCOM ≥ 3** | **PATCOM ≥ 4** |
| --- | --- | --- | --- | --- |
| **Sensitivity , %** | 98 | 89 | 61 | 16 |
| **Specificity , %** | 23 | 52 | 89 | 99 |
| **PPV, %** | 43 | 53 | 77 | 88 |
| **NPV, %** | 94 | 88 | 79 | 66 |

Legend: NPV negative predictive value; PATCOM, pulmonary artery thrombosis in COVID-19 Mallorcan score; PPV, positive predictive value
